# Effects of Melatonin on Exercise-Induced Oxidative Stress in Adults with Obesity Undergoing a Multidisciplinary Body Weight Reduction Program

**DOI:** 10.3390/jcm13175216

**Published:** 2024-09-03

**Authors:** Antonello E. Rigamonti, Federico M. Rubino, Diana Caroli, Adele Bondesan, Stefania Mai, Silvano G. Cella, Lucia Centofanti, Rita Paroni, Alessandro Sartorio

**Affiliations:** 1 Department of Clinical Sciences and Community Health, University of Milan, 20129 Milan, Italy; silvano.cella@unimi.it; 2 Department of Health Sciences, University of Milan, 20142 Milan, Italy; federico.rubino@unimi.it (F.M.R.); lucia.centofanti@unimi.it (L.C.); rita.paroni@unimi.it (R.P.); 3 Istituto Auxologico Italiano, Istituto di Ricovero e Cura a Carattere Scientifico (IRCCS), Experimental Laboratory for Auxo-Endocrinological Research, 28824 Piancavallo-Verbania, Italy; d.caroli@auxologico.it (D.C.); a.bondesan@auxologico.it (A.B.); sartorio@auxologico.it (A.S.); 4 Istituto Auxologico Italiano, Istituto di Ricovero e Cura a Carattere Scientifico (IRCCS), Laboratory of Metabolic Research, 28824 Piancavallo-Verbania, Italy; s.mai@auxologico.it

**Keywords:** obesity, melatonin, oxidative stress, inflammation, body weight reduction program, exercise

## Abstract

**Background:** Obesity is characterized by increased oxidative stress, which, in a vicious circle, promotes chronic low-grade inflammation. Melatonin, a well-documented antioxidant, might be useful as a supplement to enhance the cardiometabolic benefits of any body weight reduction program (BWRP). **Objectives/Methods:** The present study aimed to evaluate the post-exercise oxidative stress and inflammation in a group of subjects with obesity treated with melatonin (2 mg/die) or placebo, undergoing a 2-week BWRP, with the administration of a single bout of acute exercise at the start and the end of the protocol (G1–G15). **Results:** Eighteen adults with obesity were enrolled and distributed to the two arms of the study: the melatonin group (F/M: 7/2; age: 27.8 ± 5.6 years; body mass index [BMI]: 43.0 ± 4.9 kg/m^2^) and the placebo group (F/M: 6/3; age: 28.8 ± 5.0 years; BMI: 42.8 ± 4.0 kg/m^2^). BWRP induced a decrease in BMI and waist circumference (WC) in both groups; plasma glucose, blood glycated hemoglobin (HbA_1c_), and neutrophil to lymphocyte ratio (NLR) were reduced only in the placebo group. Importantly, plasma biological antioxidant potential (BAP) increased throughout BWRP. Paradoxically, melatonin enhanced post-exercise production of plasma derivatives of reactive oxygen metabolites (d-ROMs) and erythrocytic glutathionyl-Hb (HbSSG) (at G1 and G15). Finally, differently from the placebo group, melatonin-treated subjects did not exhibit the BWRP-induced decrease in plasma levels of interleukin-6 (IL-6), before and after exercise, at the end of two weeks (G15). **Conclusions:** Melatonin is presumably an antioxidant with “conditional” prooxidant actions. The use of melatonin as a supplement in subjects with obesity might be deleterious due to the abolishment of BWRP-induced cardiometabolic benefits.

## 1. Introduction

Essential obesity is characterized by increased oxidative stress, as demonstrated by high levels of some peripheral markers, such as (derivatives-) reactive oxygen metabolites (d-ROMs) [1], and by reduced antioxidant capability, which includes endogenous and exogenous antioxidant molecules and enzymatic systems endowed with antioxidant activity [2].

Notably, oxidative stress is associated with chronic low-grade inflammation, atherosclerosis with endothelial dysfunction and vasoconstriction, dysmetabolism such as insulin resistance, and cellular damage up to apoptosis, pathophysiological events that, altogether, increase the cardio-cerebro-metabolic risk in subjects with obesity [3].

Oxidative stress corresponds to the cellular damage deriving from a perturbation of the balance between pro-oxidant and anti-oxidant factors. In particular, d-ROMs are oxidant molecules generated during normal cellular metabolism when the oxygen reduction yields “unstable” free radicals [3]. d-ROMs are useful for the physiological activities within the cell, such as gene expression, cell growth, activation of the immune system, or endothelial reactivity [3,4,5]. Nevertheless, to maintain a physiologically beneficial level of d-ROMs inside the cell, there is a need for antioxidants, which, through an enzymatic or chemical mechanism, repair or prevent d-ROM-induced oxidative damage [4].

Chronic low-grade inflammation, typically present in obesity, amplifies oxidative stress. In this context, leptin, an adipose-tissue-derived peptide with predominantly central anorexigenic properties, whose plasma levels are increased in subjects with obesity, has been shown to yield oxidative stress in manifold experimental models, sustaining a vicious circle among obesity, inflammation, and oxidative stress [6,7].

Another negative element is the demonstration that intake of “obesogenic” food, particularly that enriched with saturated fats, increases oxidative stress, associated with weight gain and insulin resistance [8]. 

Reportedly, oxidative stress increases during physical exercise, a phenomenon that appears in either normal-weight subjects or subjects with obesity [9,10]. Importantly, the exercise-induced oxidative stress is increased in subjects with obesity undergoing an acute bout of endurance or resistance physical exercise. For instance, total antioxidant status (TAS) in subjects with obesity decreases by 8.6% and 17.6% in response to a single acute bout of endurance and resistance physical exercise, respectively, while there is an increase in normal-weight subjects [11]. Moreover, thiobarbituric acid reactive substances (TBARS), which represent a systemic marker of oxidative stress and lipoperoxidation (hydroperoxidation, PEROX), markedly increase in subjects with obesity undergoing physical exercise [11,12]. The comorbidity with type 2 diabetes mellitus (T2DM) further impairs the oxidative status of subjects with obesity, as demonstrated by the ensuing decrease in TAS after physical activity [13], suggesting a deleterious effect of the dysmetabolism on individual resistance to oxidative stress. The deficiency of antioxidant vitamins (A, C, and E), blood hypertension, and visceral adiposity represent further factors that can explain the prooxidant hyperresponsiveness that characterizes subjects with obesity (vs. normal-weight ones) [14].

Although the topic continues to be debated in the biomedical literature, the negative effects associated with oxidative stress might be blunted (or completely abolished?) by taking specific nutrients endowed with antioxidant activity and/or by caloric restriction.

In this context, melatonin (N-acetyl-5-methoxy-triptamine) is a lipo- and, at the same time, hydrophilic indolamine, deriving from tryptophan, produced by the pineal gland [15,16,17,18,19,20].

Different experimental studies have demonstrated that the antioxidant properties of melatonin can be both direct and indirect [17,21,22,23,24,25,26,27,28]. A “direct” action is intended to remove hydroxyl radicals, singlet oxygen, and hydrogen peroxides, and, additionally, to inhibit lipoperoxidation [15,21,27]. The melatonin-derived metabolites, formed during the scavenger activity, also possess direct antioxidant properties [15,26]. The “indirect” antioxidant action is linked to the ability of melatonin to stimulate antioxidant enzymes (e.g., superoxide dismutase, SOD; glutathione (GSH) peroxidase, GSH-Px; catalase, CAT; GSH reductase, GR) through modulation of the catalytic activity of the enzyme or regulation of the gene expression of the enzyme [15,23,25]. Melatonin is also involved in the maintenance of high levels of reduced GSH, an action that is mediated by the ability of melatonin to activate the biosynthetic pathway of GSH (e.g., stimulation of γ-glutamylcysteine synthetase [15]). 

In addition to its antioxidant properties, melatonin is endowed with anti-inflammatory activity. It removes free radicals deriving from nitric oxide (NO), a gaseous mediator involved in tissue and inflammatory processes [27]. Furthermore, melatonin inhibits the synthesis of other proinflammatory substances, including tumor necrosis factor α (TNF-α), interleukin-6 (IL-6), and interleukin-8 (IL-8) [20,24,29,30].

The pleiotropic antioxidant properties of melatonin represent a valid rationale to foresee a melatonin-based supplementation to counteract the oxidative stress that is generated during physical activity, mainly when combined with caloric restriction [31]. In particular, while a few studies carried out in elite and amateur athletes undergoing acute or chronic treatment with melatonin have evidenced an improvement in a series of peripheral markers of oxidative stress [26,31,32,33], to our knowledge, no studies have investigated the potential benefits of melatonin in subjects with obesity undergoing physical exercise (before and after caloric restriction).

The present study aimed to evaluate the exercise-induced oxidative stress in a group of adults with obesity, treated with melatonin or placebo, undergoing a 2-week body weight reduction program (BWRP), with a single bout of acute exercise at the start and the end of the protocol. Peripheral markers, such as d-ROMs and biological antioxidant potential (BAP) for oxidative stress, IL-6 and neutrophil to lymphocyte ratio (NLR) for inflammation, were measured in plasma. In addition, glutathionyl hemoglobin (HbSSG), a covalent disulfide (–S-S-) deriving from oxidative addition of glutathione to the 93-cysteine residue on the hemoglobin β-chain, was evaluated in red blood cells (RBC) as a more specific biomarker of systemic oxidative stress. 

We hypothesized that melatonin, administered to subjects with obesity, may be effective in reducing exercise-induced oxidative stress, an effect that is more evident after than before BWRP. Based on the results of the present study, as discussed below, this hypothesis will be rejected.

## 2. Materials and Methods

### 2.1. Study Design

The present clinical study was interventional and prospective, double-blind, placebo-controlled, and randomized, with the administration of two treatments (i.e., 2-week BWRP for all patients plus melatonin for the treated group, or plus placebo for the placebo group) and two exercise tests (at G1, before starting the BWRP, and G15, at the end of the BWRP). Before enrolment, patient selection was the initial phase of the study to evaluate inclusion/exclusion criteria and to obtain the patients’ consent (see below for details). 

The study protocol was approved by the Ethics Committee (EC) of the Istituto Auxologico Italiano, IRCCS, Milan, Italy (EC code: 2021_03_23_01; research project code: 01C127; acronym: MELASTRESSOB). Two drop-outs were recorded due to consent withdrawal; these subjects were excluded from the statistical analysis and replaced with two others.

### 2.2. Subjects and Protocol

Eighteen subjects with essential obesity, hospitalized at the Division of Metabolic Diseases, Istituto Auxologico Italiano, IRCCS, Piancavallo (VB), Italy, to take part in a 3-week multidisciplinary integrated BWRP, were recruited for the current study. 

The inclusion criteria were: (1) individuals of both sexes, aged ≥ 18 years; (2) individuals having a BMI > 30 kg/m^2^; (3) individuals moderately active (60 min of physical activity, two times/week); (4) female individuals in the eumenorrheic state, with the study carried out in the follicular phase of their menstrual cycle; (5) non-smokers. The exclusion criteria were: (1) secondary causes of obesity (e.g., steroid-induced obesity or Prader-Willi syndrome); (2) systolic blood pressure (SBP) ≥ 180 mmHg and diastolic blood pressure (DBP) ≥ 110 mmHg; (3) cardiovascular, psychiatric, neurological, or other (relevant) medical diseases evident in the previous 6 months; (4) intake of any drug; (5) individuals (and/or their parents) who refused to sign the consent form.

After verifying inclusion/exclusion criteria, clinical, biochemical, and anthropometric data were collected from each participant, including body composition evaluation by bioimpedance analysis (Human-IM Scan, DS-Medigroup, Milan, Italy).

Two arms of treatment were formed with a software-based randomized allocation: the first one was treated with melatonin (2 mg/die at 09.00 p.m.; Circadin, Fidia Farmaceutici, Paderno Dugnano, Milan, Italy), i.e., the melatonin group; the second one with placebo, i.e., the placebo group. For both groups, the first administration of melatonin or placebo (a tablet similar in size, color, and shape to that from Circadin without any active ingredient) was performed at G0, i.e., the day before G1, with the last one on the day before G15 (see below).

Each subject underwent, on three different days (08.30–09.30 a.m.), the following exercise protocols:

PILOT TEST (performed at G0 before starting any treatment, i.e., BWRP or melatonin/placebo). At the beginning of the study, each participant performed an incremental exercise on a treadmill (Technogym, Gambettola, Italy) until voluntary exhaustion; in particular, after 3 min of resting, the subject performed 2 min of walking at 4 km/h and 0% slope, followed by speed increments of 0.5 km/h for each min up to 6 km/h; subsequent slope increments were of 1% for each min up to 15%. Exhaustion was defined when one of the following criteria was reached: (1) maximal levels (higher than 10) of self-perceived exertion, using the Borg’s modified CR10 scale [34] or (2) heart rate (HR) values higher than 90% of the age-predicted maximum.

SUBMAXIMAL TEST (performed at G1, which corresponds to the start of the BWRP, and at G15, i.e., after two weeks of the BWRP). An exercise at a moderate constant workload, corresponding to 60% of the aerobic threshold (VO2max), established during the pilot test (see above), was maintained for 30 min or until voluntary exhaustion. 

Four blood samples, referred to as the submaximal test (at G1 and G15), were drawn from an antecubital vein of the arm by venipuncture: before exercise (basal, i.e., T0), immediately at the end of the exercise (T30), 1.5 h after (T90), and 2 h after (T150). While oxidative stress markers were performed in pre- and post-exercise time points, biochemical parameters were measured only in the basal sample (T0) (see below for details).

### 2.3. Body Weight Reduction Program (BWRP)

Though the protocol was limited to the first two weeks, the BWRP consisted of a 3-week multidisciplinary in-hospital (i.e., full-time stay in the hospital, including the night) metabolic rehabilitation, entailing an energy-restricted diet, physical exercise, psychological counseling, and nutritional education. The amount of energy to be given with diet was calculated by subtracting approximately 500 kcal from the measurement of resting energy expenditure (REE) (see below for details). The diet, in terms of macronutrients, contained approx. 21% proteins, 53% carbohydrates, and 26% lipids; the daily estimated water content was 1000 mL, while the estimated salt content was 1560 mg Na^+^, 3600 mg K^+^, and 900 mg Ca^+2^. Extra water intake of at least 2000 mL/day was encouraged. The diet was served in three meals (breakfast at 07.30 a.m., lunch at 12.30 p.m., and dinner at 07.30 p.m.). The breakfast included milk or yogurt with cereals or biscuits; the lunch was composed of a first course of pasta or rice, a second course of beef, chicken, fish or eggs with a side dish, and fruit; and the dinner included a first course of thick soup or pureed vegetables with cereals or rice, a second course of cheese, ham, fish with a side dish, and fruit. 

The physical exercise program consisted of 5 days per week of training, including (i) 1 h of dynamic aerobic standing and floor exercises with arms and legs (i.e., squats, step-ups, jump rope, lunges, push-ups, torso twists), at moderate intensity (monitored through portable cardiofrequentiometry [Polar RS400SD, Polar Electro Oy, Kempele, Finland]) and under the guidance of a therapist; and (ii) either 20–30 min cycle ergometer exercise at 60 W, or 3–4 km outdoor walking on flat terrain, according to individual capabilities and clinical status. 

The subjects also underwent a psychological counseling program (i.e., cognitive behavioral therapy strategies, such as stimulus control procedures, problem-solving, and stress management training, development of healthy eating habits, assertiveness and social skills training, cognitive restructuring of negative maladaptive thoughts, and relapse prevention training) consisting of two or three sessions per week of individual and/or group psychotherapy performed by clinical psychologists. Furthermore, lectures on the problems and risks of obesity, motivational speeches, examples of healthy foods, food preparation workshops, and group discussions, with or without a supervisor, took place daily.

### 2.4. Resting Energy Expenditure

REE was determined after an overnight fast using an open-circuit, indirect computerized calorimetry (Vmax 29, Sensor Medics, Yorba Linda, CA, USA) with a rigid, transparent, ventilated canopy. 

### 2.5. Anthropometric Measurements

A scale with a stadiometer was used to determine height (with a precision of 0.1 cm) and weight (with a precision of 0.1 kg) (Wunder Sa.Bi., WU150, Trezzo sull’Adda, Italy). Waist circumference (WC) was measured with a flexible tape in a standing position, halfway between the inferior margin of the ribs and the superior border of the crista, while hip circumference (HC) was measured at the largest parts around the buttocks. Body composition was measured by bioimpedance analysis (Human-IM Scan, DS-Medigroup, Milan, Italy) after 20 min of supine resting. BMI (weight in kg divided by height in meters squared), fat mass (FM), and fat-free mass (FFM) were determined in all subjects. 

### 2.6. Biological Sample Collection

Blood samples were collected from patients following a standardized protocol (at T0, T30, T90, and T150 for G1 and G15). The same types of tubes and consumables for each cluster of parameters were used throughout the entire duration of the study to improve consistency. 

Blood samples were collected in lithium heparin tubes after an overnight fast. Serum tubes were also used. Cells were separated from plasma/serum by centrifugation (20–24 °C for 10 min at 2500× *g*) within 2 h from the blood collection. Blood cells (mainly red blood cells, RBC) and plasma were then transferred in separated pre-cooled tubes and put in ice to preserve from degradation oxidative stress markers. Each plasma/serum-containing tube was vortexed for at least 10 s, divided into aliquots, and stored at −20 °C.

Half of the plasma/serum samples and RBC samples were delivered from Piancavallo-Verbania to Milan while keeping the samples frozen, where they were stored at −20 °C until the analyses for markers of oxidative stress.

### 2.7. Metabolic, Biochemical and Hormonal Evaluation

Total cholesterol (T-C), high-density lipoprotein cholesterol (HDL-C), low-density lipoprotein cholesterol (LDL-C), triglycerides (TG), glucose, insulin, IL-6, and high-sensitivity C-reactive protein (hs-CRP) were measured.

Colorimetric enzymatic assays (Roche Diagnostics, Monza, Italy) were used to determine serum T-C, LDL-C, HDL-C, and TG levels. The sensitivities of the assays were 3.86 mg/dL [1 mg/dL = 0.03 mmol/L], 3.87 mg/dL [1 mg/dL = 0.03 mmol/L], 3.09 mg/dL [1 mg/dL = 0.03 mmol/L], and 8.85 mg/dL [1 mg/dL = 0.01 mmol/L], respectively.

Serum glucose level was measured using the glucose oxidase enzymatic method (Roche Diagnostics, Monza, Italy). The method’s sensitivity was 2 mg/dL [1 mg/dL = 0.06 mmol/L]. Serum insulin concentration was determined by a chemiluminescent immunometric assay, using a commercial kit (Elecsys Insulin, Roche Diagnostics, Monza, Italy). The sensitivity of the method was 0.2 µU/mL [1 µU/mL = 7.18 pmol/L].

The intra- and inter-assay coefficients of variation (CVs) were: 1.1% and 1.6% for T-C, 1.2% and 2.5% for LDL-C, 1.8% and 2.2% for HDL-C, 1.1% and 2.0% for TG, 1.0% and 1.3% for glucose, and 1.5% and 4.9% for insulin.

Values of hs-CRP were determined by a particle-enhanced immunoturbidimetric test (Roche Diagnostics GmbH, Mannheim, Germany). The sensitivity was 0.15 mg/L (1.43 nmol/L). 

Plasma levels of IL-6 were measured by ECLIA (Roche Diagnostics GmbH, Mannheim, Germany), which has a sensitivity of 1.5 pg/mL. 

For each patient, the homeostatic model assessment of insulin resistance (HOMA-IR) was also calculated using the formula (insulin [μU/mL] × glucose [mmol/L])/22.5 [35].

### 2.8. Measurement of Blood Pressure

Blood pressure was measured on the right arm, using a sphygmomanometer with an appropriate cuff size, with the subject in a seated and relaxed position. The procedure was repeated three times at 10-min intervals; the means of the three values for systolic (SBP) and diastolic (DBP) blood pressure were recorded.

### 2.9. Analytical Methods for d-ROMs and BAP

Serum d-ROMs were measured as previously described [36,37]. Briefly, the spectrophotometric test, based on a commercial kit (Diacron International, Grosseto, Italy), measures the “oxidizing” capacity of a plasma sample against a particular substance (a modified aromatic amine) used as an indicator (chromogen). The phenomenon is associated with the gradual and progressive change towards the rose (505 nm) of the reaction mixture (plasma + chromogen), initially colorless. The oxidizing capacity measured by d-ROMs is mainly due to in vitro alcoholic and hydroperoxyl radicals, derived from in vivo formed hydroperoxides (ROOH) in the presence of transition metals acting as catalysts. The resulting d-ROMs values were obtained in arbitrary units (U.Carr., Carratelli Units), then converted into mmol/L of H_2_O_2_, as 1 CARR U is stated by the manufacturer to be equivalent to 0.08 mg/dL. The sensitivity of the d-ROMs test was 0.26 mM H_2_O_2_, and the method was linear up to 267 mmol/L. Intra- and inter-assay CVs were 2.07% and 1.79%, respectively.

When free radicals react with a correctly buffered chromogenic substance, they develop a colored complex. The concentration of the colored complex is directly proportional to the concentration of hydroperoxides. 

Serum BAP was measured by a spectrophotometric method using the OXY-Adsorbent test commercial kit on a FREE analyzer (Diacron International, Grosseto, Italy), as previously described [38]. The test is based on the ability of hypochlorous acid (HClO) to oxidize the physiological antioxidants (uric acid, glutathione, thiol groups, vitamins, glutathione peroxidase, superoxide dismutase, and catalase). As HClO reacts with a correctly buffered chromogenic substrate, it forms a colored complex that can be measured photometrically at 505 or 546 nm. The concentration of the colored complex is directly proportional to the concentration of HClO and indirectly proportional to the antioxidant ability of the sample. The analytical imprecision of the test is: CV within-run = 1.90%; CV between-run = 2.05%.

### 2.10. Measurement of HbSSG by MALDI

The measurement of HbSSG was accomplished by MALDI-ToF mass spectrometry, according to the recently published analytical method in a Bruker Autoflex II instrument [39]. Briefly, the technique uses sinapinic acid (0.1 M in 0.1% TFA-acetonitrile 1:1 *v*/*v*) as the MALDI matrix and a 10-microM titrated RBC hemolysate. Batches of 24 (2), 48 (1), or 72 (1) samples were analyzed within 24 h from preparation from thawed RBC cells. For each sample, four 1-microliter instrumental replicates were measured, and the results were analyzed from the obtained binary mass-intensity files in a custom Microsoft Excel^®^ (Microsoft 365) worksheet. Glutathionyl-hemoglobin level in the samples is calculated as the ratio of the integrated areas of the single-charged (MH+) species of beta-Hb and HbSSG in each of the four replicate measurements, and the calculation spreadsheet yields the corrected percent ratio value and the corresponding standard error. The standard error in the 141-sample group is below 0.1%.

### 2.11. Statistical Analysis

The SigmaPlot 14.0 statistical software package was used for data analyses, while GraphPad Prism 9.0 software was used for plotting data.

Before starting patient recruitment, as requested by the Ethics Committee, we calculated the sample size: in particular, eighteen subjects (i.e., 9 × 2) were adequate to observe a mean difference of d-ROMs plasma levels equal to 50.0 ± 35.0 U.Carr between the two experimental groups (BWRP + placebo vs. BWRP + melatonin at the end of the intervention), applying a two-tailed *t*-test for unpaired data, with a *p*-value < 0.05 and a power of 0.80.

The Shapiro–Wilk test showed that all parameters were normally distributed.

Results are reported as mean ± standard deviation (SD). The post-exercise responses in d-ROMs, BAP, HbSSG, and IL-6 were evaluated as absolute values for each experimental group (melatonin vs. placebo).

Demographic, biochemical, and clinical parameters were compared within each experimental group (melatonin or placebo) before (G1) and after (G15) BWRP (intragroup analysis) and between the two experimental groups (melatonin vs. placebo) for G1 (before BWRP) or G15 (after BWRP) (intergroup analysis) using a two-way ANOVA (with the two factors “treatment” [melatonin/placebo] × “BWRP” [G1–G15]), followed by Bonferroni’s post hoc test. 

In this first tier of results elaboration, parameters of oxidative stress (d-ROMs, BAP, HbSSG, and IL-6) were compared taking into account groups of treatment, BWRP, and exercise by using a three-way ANOVA (with the three factors “treatment” [melatonin/placebo] × “exercise” [T0–T30 for d-ROMs, BAP and IL-6 or T0–T30–T90–T150 for HbSSG] × “BWRP” [G1–G15]), followed by Bonferroni’s post hoc test. 

Significance was set at a level of *p* < 0.05 for all data analyses.

## 3. Results

Table 1 reports the results of a series of two-way ANOVAs with “treatment” (melatonin/placebo) × “BWRP” (G1/G15), aimed at comparing demographic, clinical, and biochemical parameters. 

In short, BMI and WC significantly decreased in the melatonin and placebo groups at the end of the two weeks of BWRP (*p* < 0.05). The beneficial effect of BWRP on BMI and WC did not depend upon treatment with melatonin. 

In contrast to from the unchanged values of SBP, without considering the factor “treatment”, DBP significantly decreased at G15, i.e., the end of the two weeks of BWRP (vs. G1, *p* < 0.05), a difference that was missed when comparing the effect of BWRP within each group of treatment, i.e., melatonin or placebo. While BWRP failed to change HR in the placebo group, there was a BWRP-induced decrease in HR among subjects belonging to the melatonin group (G15 vs. G1, *p* < 0.05).

Serum levels of glucose and the percentage of HbA_1c_ were significantly reduced by BWRP only in the placebo group (G1 vs. G15, *p* < 0.05), without any effect of the treatment (melatonin/placebo). A similar effect of BWRP (but not treatment) was observed on NLR, which decreased only in the placebo group (G1 vs. G15, *p* < 0.05).

**Figure 1:**
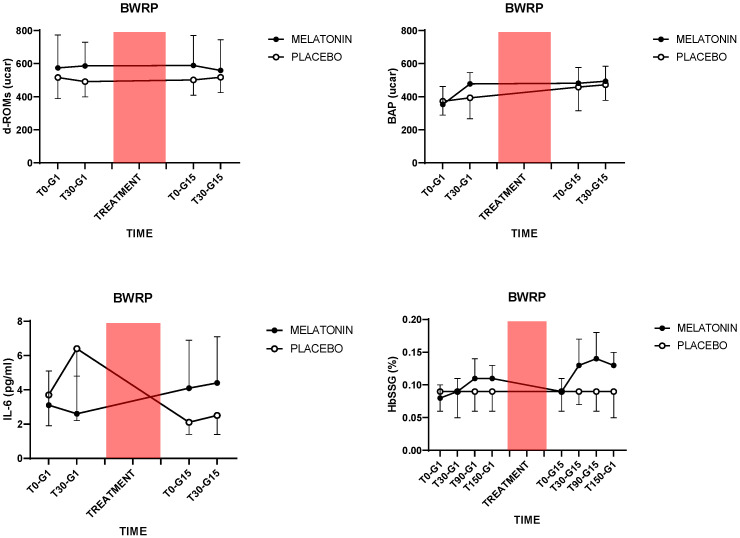
Post-exercise plasma levels of d-ROMs (top left panel), BAP (top right panel), and IL-6 (bottom left panel), and erythrocytic levels of HbSSG (bottom right panel) in adults with obesity, treated with melatonin (2 mg/die) or placebo, undergoing a 2-week BWRP, with a single bout of acute exercise at the start and the end of the protocol. The colored box indicates the duration of treatment and BWRP (i.e., two weeks). The first dose of melatonin was taken at G0 (in the evening before starting the BWRP). The interval T0-T30 corresponds to the duration of the exercise test, while T90 and T150 are post-exercise time points. For abbreviations, see the text. For statistical analysis, see the Section 2.11.

Applying a three-way ANOVA (treatment [melatonin-placebo] × BWRP [G0–G15] × exercise [T0–T30], when evaluating plasma levels of d-ROMs, only the factor “treatment” was statistically significant, indicating that treatment with melatonin increased plasma production of d-ROMs (melatonin vs. placebo: Δ = 70.306 ucarr; t = 2.054; *p* = 0.044) (Figure 1; Table 2). 

Moreover, only the factor “BWRP” was found to be capable of explaining the statistically significant increase in plasma BAP at the end of the two weeks (G1 vs. G15: Δ = 76.8283 ucarr; t = 144; *p* = 0.003) (Figure 1; Table 3). 

When evaluating plasma levels of IL-6, a statistically significant interaction was found between the factors “treatment” × “BWRP” (*p* < 0.001). In particular, independently from exercise (T0 vs. T30), plasma levels of IL-6 significantly decreased in the placebo group (G1 vs. G15: Δ = 2.772 pg/dL; t = 3.435; *p* < 0.001), but not in the melatonin group (G15 vs. G1: Δ = 1.394 pg/dL; t = 1.728; *p* = 0.089). Furthermore, independently from exercise (T0 vs. T30), at G1, plasma levels of IL-6 were significantly higher in the placebo than in the melatonin group (placebo vs. melatonin: Δ = 2.200 pg/dL; t = 2.726; *p* = 0.008), being significantly lower at G15 (melatonin vs. placebo: Δ = 1.967 pg/dL; t = 2.437; *p* = 0.018) (Figure 1; Table 4).

Applying a three-way ANOVA (treatment [melatonin-placebo] × BWRP [G0–G15] × exercise [T0–T30–T90–T150], when evaluating erythrocytic levels of HbSSG, the factor “treatment” was statistically significant, indicating that treatment with melatonin increased erythrocyte production of HbSSG (melatonin vs. placebo: Δ = 0.0228 ucarr; t = 4.611; *p* < 0.001). Due to the existence of a statistically significant interaction between the factors “treatment” × “exercise”, the exercise-induced effects on HbSSG were evaluated for each group of treatment (i.e., melatonin and placebo): in particular, within the melatonin group, erythrocytic levels of HbSSG were significantly higher at T90 (Δ = 0.0374%; t = 3.747; *p* = 0.002) and T150 (Δ = 0.0322%; t = 3.232; *p* = 0.009) vs. T0, whereas, within the placebo group, exercise induced no significant difference in erythrocytic HbSSG for any time vs. T0. Moreover, when evaluating the comparisons for the factor “treatment” (i.e., melatonin and placebo) within the different time points of the exercise test, erythrocytic levels of HbSSG were significantly higher in the melatonin than in the placebo group either at T30 (Δ = 0.0233%; t = 2.376; p = 0.019) or T90 (Δ = 0.0368%; t = 3.691; *p* < 0.001). Finally, independently from the other factors, i.e., “treatment” and “exercise”, BWRP induced a significant effect on erythrocytic levels of HbSSG, which were significantly higher at G15 than at G1 (Δ = 0.0120%; t = 2.421; *p* = 0.017) (Figure 1; Table 5).

## 4. Discussion

Based on the results obtained in the present study, carried out in a group of subjects with obesity, treated with melatonin or placebo undergoing a BWRP with a single bout of acute exercise at the start and the end of the protocol, we can summarize as follows:

(1) Two weeks of BWRP, combined with a placebo (i.e., without any “active” treatment with melatonin), exerted anti-inflammatory effects, as demonstrated by the decreases in plasma IL-6 and blood NLR; the former a pro-inflammatory cytokine [40] and the latter a peripheral biomarker that conjugates two components of the immune system, i.e., the innate immune response, mainly due to neutrophils, and adaptive immunity, supported by lymphocytes [41]. Parallelly, when measuring a surrogate of antioxidant capabilities in the organism [42], a BWRP-induced increase in plasma BAP occurred, an effect that was independent of the treatment with melatonin. Melatonin, independently from BWRP, increased plasma d-ROMs and erythrocytic HbSSG, two peripheral markers of oxidative stress [43], which were generated in each exercise test (at G1 and G15 for T90 and T150);
(2) Melatonin, when acutely administered (i.e., the single dose at G0), reduced plasma levels of IL-6 at G1 (T0), implying a different anti-inflammatory vs. pro-inflammatory effect for single or continuous administration;
(3) Apart from decreases in BMI and WC that were found in both groups, BWRP exerted additional metabolic benefits only in the placebo group (e.g., decreases in plasma glucose and blood HbA1c).

Reportedly, obesity represents a condition of increased oxidative stress that, pathophysiologically, in a vicious circle, promotes several obesity-associated cardiometabolic diseases, such as blood hypertension, T2DM, atherosclerosis, chronic low-grade inflammation, endothelial dysfunction, etc. [44]. Interestingly, interventions aimed at reducing body weight, particularly diet combined with exercise, have been demonstrated to tone down obesity-related oxidative stress, a beneficial effect indirectly established by evaluating markers of oxidative stress or antioxidant potential in the plasma [14,45].

In the present study, perhaps due to the short duration of the BWRP (i.e., two weeks), there were no decreases in plasma d-ROMs and erythrocyte HbSSG in placebo-treated subjects. Nevertheless, a progressive increase in plasma BAP occurred, an effect related to the BWRP but independent from treatment with melatonin. In opposition to the rise in plasma BAP, melatonin, a well-recognized antioxidant agent [46], in the present study, paradoxically, stimulated post-exercise production of plasma d-ROMs and erythrocyte HbSSG. Importantly, exercise *per se* failed to stimulate plasma d-ROMs and erythrocyte HbSSG in the placebo group.

To our best knowledge, no one has so far demonstrated a prooxidant effect of melatonin in a clinical context, with only in-vitro data being available (e.g., tumor cells) [47]. A (fascinating) interpretation of these conflicting results is admitting that melatonin is a “well-documented antioxidant with conditional prooxidant actions”—as reported in the title of a recent review on this topic [47]—indicating that melatonin is primarily an antioxidant agent but, when tested under specific experimental conditions (i.e., conditionally), becomes prooxidant (the “Zhang & Zhang” effect). In this regard, some authors have invoked an interference of melatonin with mitochondrial function, with ensuing hyperproduction of free radicals from the respiratory chain [48].

The dose-response antioxidant effect of melatonin has been studied in animal models [49] and in ex-vivo human cellular systems [50]; in both cases, non-linear, “reverse-U-shaped” hormetic dose-dependence has been observed [51]. Human RBCs were studied both in vitro and in vivo, also with complementary human-rat experiments [52,53,54]; an antioxidant (protective) effect was observed in oxidatively stressed RBCs at nanomolar concentrations of melatonin; however, at millimolar levels, prooxidant (deleterious) effects took place [52]. This phenomenon, i.e., the concentration-dependence of melatonin effects on RBCs, is still poorly understood at the molecular level. Importantly, when assessing antioxidant-prooxidant effects of melatonin on RBCs, one should be aware that results obtained in rodents [54] are not directly comparable to those in humans, since rat hemoglobin shows very different response-properties towards oxidants due to the presence of an additional cysteine (endowed with a -SH group) residue in the primary structure of the protein [55].

On the other hand, we prefer underscoring the factors of our experimental protocol that would have conditionally revealed the melatonin-mediated prooxidant actions, among which are obesity, caloric restriction (i.e., BWRP), (acute) exercise, and dose/duration of melatonin treatment (i.e., relatively low dose and short-term treatment). In particular, despite the BWRP-induced BAP restoration over the two weeks, the molecular pathways underlying the direct and indirect antioxidant effects of melatonin appear to be blocked, allowing the melatonin-induced prooxidant actions to emerge.

This view of intending melatonin endowed with both antioxidant and prooxidant actions [47] is congruent with the other results obtained in the present study. Plasma levels of IL-6 were, at the end of the two weeks, higher in the melatonin group than in the placebo group, suggesting that treatment with melatonin had abolished the beneficial BWRP-induced reduction of the inflammatory state, which, instead, was documented in the placebo group [56]. As a link has been shown between oxidative stress and inflammation [57], particularly in subjects with obesity [44], we argue that melatonin, due to its own (conditional) prooxidant actions [47], could become deleterious in subjects with obesity when combined with caloric restriction, particularly under conditions of acute exercising. Interestingly, in accordance with our view of an “antioxidant/prooxidant” melatonin, the antioxidant or, more correctly, anti-inflammatory action of melatonin could be observed only at the start of our protocol, when the main conditioning factors were missing (e.g., BWRP and exercise), i.e., T0 at G1.

Differently from the placebo group, the missing statistical significance of BWRP-induced decreases in plasma glucose, blood HbA1c, and NLR in the melatonin group might be useful to reinforce our interpretation of the data. When fully expressed, the prooxidant nature of melatonin may abolish the cardiometabolic benefits deriving from weight loss. Further studies enrolling a larger number of subjects are mandatory to statistically consolidate these preliminary results and arguments.

In addition to the previous arguments, we would like to explain our results according to the modern theory of a dynamic redox equilibrium, oscillating from oxidative stress to reductive stress, a process that is essential for cellular homeostasis but that, when altered (by melatonin or other antioxidant compounds?), can be deleterious [58]. In particular, as previously described, overproduction of d-ROMs and/or depletion of antioxidant enzymatic and non-enzymatic systems may lead to oxidative stress. On the contrary, an excessive increase in reducing equivalents such as reduced glutathione (i.e., increased GSH/GSSG ratio) and reduced NADPH—a state that presumably occurs at the start of melatonin treatment—can eliminate all d-ROMs, driving to reductive stress. When a long-lasting reductive stress is present—e.g., the continuation of melatonin treatment for two weeks—we guess an oxidative stress is induced by a feedback mechanism [59,60]. Indeed, during reductive stress, when electron acceptors are expected to be mostly reduced, some redox proteins can donate electrons to O_2_, thus generating additional superoxide that is unmatched by inducible SOD and thus produces d-ROMs [61]. Immediately afterward, a high level of reducing equivalents enhances d-ROMs scavenging systems, involving redox couples such as the NAD/NADH^+^, NADPH/NADP^+^, and GSH/GSSG ratio [61,62], resulting in a net H_2_O_2_ spillover from mitochondria that favors reductive stress, which, in its turn, stimulates oxidative stress [62]. This (fascinating) dynamism of the redox equilibrium might explain why chronic consumption of antioxidant supplements, such as vitamins and/or flavonoids, has been associated with prooxidant effects due to a perturbation of the cellular redox equilibrium, which contributes to reactive stress, even causing inflammation (such as obesity) and diminishing life expectancy in animals and humans [58].

For what concerns the RBCs, where glutathionyl-hemoglobin is measured as a biomarker of compartment-specific oxidative stress, it should be considered that mature, circulating erythrocytes do not possess cellular machinery on which melatonin can exert induction of antioxidant enzyme production, and circulating levels (expressed as intra-erythrocyte melatonin pool) do not justify antioxidant actions on a stoichiometric basis, i.e., total glutathione and HbSSG pool is in the high nanomolar-low micromolar range. It is conceivable that temporary or definitive partial inactivation of the pool of erythrocyte antioxidant enzymes by some of the ROS, especially those that are measured as d-ROMs or TBARS, explains the lack of or delayed reduction of HbSSG and its raised level in the subjects.

Before closing, some limitations of our study should be mentioned. First of all, we have used only one dose of melatonin, which might exhibit different properties, even opposed (pro- vs. anti-oxidant), when low vs. high doses are administered. Second, a limited number of subjects were recruited, but the complexity of the protocol discouraged some subjects from participating in the study at the selection time; thus, we could have missed some statistically significant results. Third, we have not measured plasma levels of melatonin. However, in our experimental context, taking also into account its short half-life, we were primarily interested in its biological effects.

## 5. Conclusions

Melatonin administration is not recommended in subjects with obesity undergoing a BWRP because it promotes oxidative stress and inflammation, which are generally reduced by interventions for weight loss [45]. We suggest that the use of antioxidants as a supplement in subjects with obesity should be guided by the results of well-designed clinical studies, which might evidence contrasting effects under different conditions [63].

## Figures and Tables

**Table 1:** Demographic, biochemical, and clinical characteristics of the study population, including subjects with obesity undergoing a 2-week BWRP (G1-G15), treated with melatonin (2 mg/die) or placebo.

Parameter	Melatonin		Placebo	
	G1	G15	G1	G15
N.	9	9	9	9
Sex (F/M)	7/2	7/2	6/3	6/3
Age (years)	27.8 ± 5.6	27.8 ± 5.6	28.8 ± 5.0	28.9 ± 5.0
BMI (kg/m^2^)	43.0 ± 4.9	41.6 ± 4.4 ^a^	42.8 ± 4.0	40.7 ± 4.0 ^b^
WC (cm)	110.2 ± 10.0	104.6 ± 9.5 ^a^	119.1 ± 12.1	111.3 ± 11.5 ^b^
SBP (mmHg)	131.1 ± 7.8	122.2 ± 8.3	122.9 ± 14.0	123.9 ± 10.5
DBP (mmHg)	81.1 ± 6.0	77.2 ± 4.4	79.4 ± 8.8	75.6 ± 7.7
HR (bpm)	92.1 ± 13.3	81.3 ± 7.2 ^a^	83.7 ± 12.6	80.3 ± 14.5
FFM (kg)	55.1 ± 7.2	54.1 ± 6.7	50.0 ± 21.1	49.4 ± 21.5
FFM (%)	47.8 ± 4.7	48.2 ± 4.6	51.4 ± 7.0	50.9 ± 9.0
FM (kg)	60.4 ± 8.8	58.3 ± 8.4	49.9 ± 20.2	49.0 ± 20.4
FM (%)	52.2 ± 4.7	51.8 ± 4.6	48.6 ± 7.0	48.6 ± 8.1
Glucose (mg/dL)	88.3 ± 8.8	86.4 ± 6.9	87.1 ± 6.2	82.3 ± 6.1 ^b^
Insulin (mU/L)	17.8 ± 6.0	19.1 ± 7.4	18.3 ± 6.9	17.7 ± 5.9
HOMA-IR	3.9 ± 1.4	4.1 ± 1.1	4.0 ± 1.7	3.6 ± 1.4
T-C (mg/dL)	148.7 ± 28.3	141.9 ± 31.5	166.2 ± 33.9	160.1 ± 21.2
HDL-C (mg/dL)	46.0 ± 10.3	45.7 ± 11.3	39.7 ± 8.4	40.4 ± 9.2
LDL-C (mg/dL)	85.4 ± 28.6	77.7 ± 28.0	106.0 ± 30.4	99.2 ± 17.5
TG (mg/dL)	102.9 ± 43.7	111.9 ± 47.0	145.3 ± 77.3	123.6 ± 43.3
HbA_1c_ (%)	5.2 ± 0.5	5.2 ± 0.5	5.3 ± 0.3	5.1 ± 0.3 ^b^
hs-CRP (mg/dL)	0.56 ± 0.4	0.69 ± 0.9	0.50 ± 0.4	0.3 ± 0.3
NLR	1.6 ± 0.4	1.5 ± 0.4	1.7 ± 0.3	1.3 ± 0.4 ^b^

^a^: *p* < 0.05 for the factor “BWRP” within the melatonin group (i.e., factor “treatment”). ^b^: *p* < 0.05 for the factor “BWRP” within the placebo group (i.e., factor “treatment”). Abbreviations: BMI, body mass index; DBP, diastolic blood pressure; FFM, fat-free mass; FM, fat mass; HbA_1c_, glycated hemoglobin; HDL-C, high-density lipoprotein cholesterol; HOMA-IR, homeo-stasis model assessment of insulin resistance; HR, heart rate; hs-CRP, high-sensitivity C-reactive protein; hemoglobin; LDL-C, low-density lipoprotein cholesterol; NLR, neutrophil to lymphocyte ratio; SBP, systolic blood pressure; T-C, total cholesterol; TG, triglycerides; WC, waist circumference.

**Table 2:** Report of all pairwise multiple comparison procedures (Bonferroni *t*-test), related to the three-way ANOVA of plasma levels of d-ROMs, with the following factors: treatment (melatonin-placebo), BWRP (G1–G15) and exercise (T0–T30).

Comparison	Diff. of Means	*t*	*p*
*Comparisons for factor: TREATMENT*			
MELATONIN vs. PLACEBO	70.306	2.054	0.044
*Comparisons for factor: EXERCISE*			
T0 vs. T30	7.083	0.207	0.837
*Comparisons for factor: BWRP*			
G1 vs. G15	0.361	0.0105	0.992

**Table 3:** Report of all pairwise multiple comparison procedures (Bonferroni *t*-test), related to the three-way ANOVA of plasma BAP, with the following factors: treatment (melatonin-placebo), BWRP (G1–G15) and exercise (T0–T30).

Comparison	Diff. of Means	*t*	*p*
*Comparisons for factor: TREATMENT*			
MELATONIN vs. PLACEBO	27.462	1.124	0.265
*Comparisons for factor: EXERCISE*			
T30 vs. T0	42.664	1.746	0.086
*Comparisons for factor: BWRP*			
G15 vs. G1	76.828	3.144	0.003

**Table 4:** Report of all pairwise multiple comparison procedures (Bonferroni *t*-test), related to the three-way ANOVA of plasma levels of IL-6, with the following factors: treatment (melatonin-placebo), BWRP (G1–G15) and exercise (T0–T30).

Comparison	Diff. of Means	*t*	*p*
*Comparisons for factor: TREATMENT*			
PLACEBO vs. MELATONIN	0.117	0.204	0.839
*Comparisons for factor: EXERCISE*			
T30 vs. T0	0.739	1.295	0.200
*Comparisons for factor: BWRP*			
G1 vs. G15	0.689	1.207	0.232
*Comparisons for factor: BWRP within PLACEBO*			
G1 vs. G15	2.772	3.435	0.001
*Comparisons for factor: BWRP within MELATONIN*			
G15 vs. G1	1.394	1.728	0.089
*Comparisons for factor: TREATMENT within G1*			
PLACEBO vs. MELATONIN	2.200	2.726	0.008
*Comparisons for factor: TREATMENT within G15*			
MELATONIN vs. PLACEBO	1.967	2.437	0.018

**Table 5:** Report of all pairwise multiple comparison procedures (Bonferroni *t*-test), related to the three-way ANOVA of intra-erythrocytic levels of HbSSG, with the following factors: treatment (melatonin-placebo), BWRP (G1–G15) and exercise (T0–T30–T90–T150).

Comparison	Diff. of Means	*t*	*p*
*Comparisons for factor: TREATMENT*			
MELATONIN vs. PLACEBO	0.0228	4.611	<0.001
*Comparisons for factor: EXERCISE*			
T90 vs. T0	0.0184	2.630	0.058
T90 vs. T30	0.0101	1.439	0.916
T90 vs. T150	0.00285	0.404	1.000
T150 vs. T0	0.0156	2.223	0.168
T150 vs. T30	0.00722	1.032	1.000
T30 vs. T0	0.00833	1.200	1.000
*Comparisons for factor: BWRP*			
G15 vs. G1	0.0120	2.421	0.017
*Comparisons for factor: EXERCISE within MELATONIN*			
T90 vs. T0	0.0374	3.747	0.002
T90 vs. T30	0.0168	1.685	0.566
T90 vs. T150	0.00514	0.508	1.000
T150 vs. T0	0.0322	3.232	0.009
T150 vs. T30	0.0117	1.170	1.000
T30 vs. T0	0.0206	2.093	0.230
*Comparisons for factor: EXERCISE within PLACEBO*			
T0 vs. T30	0.00389	0.396	1.000
T0 vs. T150	0.00111	0.113	1.000
T0 vs. T90	0.000556	0.0566	1.000
T90 vs. T30	0.00333	0.339	1.000
T90 vs. T150	0.000556	0.0566	1.000
T150 vs. T30	0.00278	0.283	1.000
*Comparisons for factor: TREATMENT within T0*			
PLACEBO vs. MELATONIN	0.00111	0.113	0.910
*Comparisons for factor: TREATMENT within T30*			
MELATONIN vs. PLACEBO	0.0233	2.376	0.019
*Comparisons for factor: TREATMENT within T90*			
MELATONIN vs. PLACEBO	0.0368	3.691	<0.001
*Comparisons for factor: TREATMENT within T150*			
MELATONIN vs. PLACEBO	0.0322	3.232	0.002

## Data Availability

The datasets used and/or analyzed in the present study are available from the corresponding author upon reasonable request. Raw data will be uploaded to www.zenodo.org immediately after the manuscript is accepted.

## Abbreviations

BAP: biological antioxidant potential; BMI, body mass index; BWRP, body weight reduction program; DBP, diastolic blood pressure; diff, difference; d-ROMs, derivate-reactive oxygen metabolites; EC, Ethical Committee; FFM, fat-free mass; FM, fat mass; GSH, glutathione; GSH-Px, glutathione peroxidase; HbA1c, glycated hemoglobin; HbSSG, glutathionyl hemoglobin; HC, hip circumference; HDL, high density lipoprotein; HOMA-IR, homeostasis model assessment of insulin resistance; HR, heart rate; hs-CRP, high sensity C-reactive protein;IDF, International Diabetes Federation; IL-6, interleukin-6; IL-8, interleukin-8; LDL, low-density lipoprotein; NADH, nicotinammide adenina dinucleotide; NLR, neutrophils to lymphocyte ratio; NO, nitric oxide; PEROX, hydroperoxidation; RBC, red blood cell; REE, resting energy expenditure; SBP, systolic blood pressure; SOD, superoxide-dismutase; T2DM, type 2 diabetes mellitus; TAS, total antioxidant status; TBARS, thiobarbituric acid reactive substances; T-C, total cholesterol; TG, triglycerides; TNF-α, tumor necrosis factor α; WC, waist circumference.
